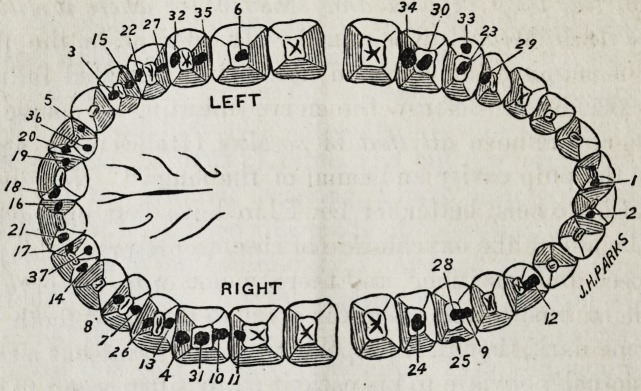# Exposed Pulps

**Published:** 1872-02

**Authors:** Arthur J. Ford

**Affiliations:** Atlanta, Ga.


### AMERICAN JOURNAL

OF

### DENTAL SCIENCE.

Vol. V. THIRD SERIES--FEBRUARY, 1872. No. 10.

### ARTICLE I.

### *Exposed Pulps.*

BY ARTHUR J. FORD, D. D. S., of Atlanta, Ga.

I have for years been much interested and exercised in every means devised, and all operations tending towards the preservation of the vitality of exposed pulps, and have ever greedily caught at every proposed method that was in the least feasible or that pointed towards the attainment of this desideratum, and have as anxiously hoped to at last alight upon some certain and reliable remedy for the great and crying evil of the wholesale devitalization of dental pulps, as ever did the alchemists of old, yearn to discover the "Philosopher's Stone," and I believe we have met with greater success than the above named ancient gentlemen, and I feel almost constrained to exclaim "Eureka," at the results attained by the use of oxychloride of zinc or "Cement Plombe," and kindred preparations for this laudable purpose. At all events I speak for myself when I say that I have had more uniform and fortuitous results, from the careful and judicious use of this article (Cement Plombe) than from any former operation devised, as a successful protector of this extremely sensitive organ.

I herewith transmit to you a carefully kept register of 78 of such operations performed by myself, with the results, within the past twenty months, together with a diagram exhibiting the position of every cavity treated, and with marginal notes where particular cases are indicated; all not specially marked are doing well as far as heard from. The large majority I have seen recently and many I meet almost daily, most are regular patients of mine, and but few accidental. I think I can say without boasting, I have been as successful as most that have tried this mode of operating, and considering the size of our community, I have had quite a large proportion of subjects. By the register appended, you will perceive that lately the cases have increased quite rapidly, having filled this year to date, (December 15th,) 62, against 16 for last year. I consider this due to the success I have had in this mode of treatment. Prior to my adopting this capping, I used to have almost constantly from two to eight devitallized teeth on hand to be treated, and pulp cavities to be filled, which was arduous to the operator and fatiguing to the patient. Now I rarely destroy a pulp, and never except in extreme cases. I could give quite a number of cases similarly treated, anterior to the first date of this register, as I have been gradually adopting this practice since June 1868, but the cases were so scattering that I kept no certain record of them, but some that I know were so filled in 1868 are perfectly successful.

I have been lead to the consideration of this subject, and to come forward as one of the champions of this mode of treatment, from the fact that I have heard eminent men in our profession, quite recently condemn it, and express many doubts of its being permanently successful, even under the most favorable circumstances, and having, as I believe, been successful in a large number of cases, for the period of at least two, and in some instances nearly four years, I most heartily tender my abuttal testimony.

Since writing the above I have received the "Dental Cosmos," wherein I find two articles, one from the pen of Dr.

S. B. Palmer, of Syracuse, N. Y., a gentleman of known eminence in our profession, wherein he undertakes to fill a tooth, "*the pulp exposed and sensitive*, or *where it still retains its vitality*, (Italics mine.) He casts aside the practice of capping the pulp, on account of repeated failures, and decides to destroy the nerve (meaning of course the pulp) and remove *all that is possible*, (Italics mine again) from the pulp cavity and canal of the fangs." Now would it not have been better for Dr. P. to have first endeavored by the aid of the oxychloride of zinc, to preserve "this pulp exposed and sensitive," and thereby not only preserve the tooth in health and *color* (for every devitallized tooth will become dark, though if properly treated I grant but a trifle) but actually obviate to his patient many sittings and to himself, the operator, an extended, frequently anxious, and often tedious operation; again, he admits the possibility of not being able to remove entirely in all cases all of the pulp, (upper bicuspids and molars I presume) and in such cases a failure is possible to say the least; whereas if the capping is not successful, he still has left him the "dernier resort" of extirpation. I have had only two such cases that resisted capping, one of a central incisor and the other a superior molar; I devitalized both and have saved them to date. Both these cases were hardly fair tests, as they had been maltreated prior to my taking them in hand.

The other article is from Dr. Redesicks, of Iowa, who demonstrates by a series of experiments the many difficulties to be overcome in extirpating pulps *entirely*, and filling canals, which speaks to my mind, volumes in favor of capping, if successful, and I think I can show it has been with me, and therefore can be with any careful operator, thereby obviating all the worry and likelihood of ultimate failure.

Some eminent practitioners aver, that the pulp is not preserved by this process of capping, but is finally destroyed and taken up by the absorbents and the canal is left vacant but causing no trouble. This I deny, but even granting it true, if the tooth is retained in ease and usefulness, is not the practice commendable, and the desired result attained?

The following is the diagram and register above alluded to:

1870.

April 29th, Mr. J., No. 32. May 17th, Miss E. C., No. 13. May 24th, Mrs. R., No. 32. May 25th, Miss L. No. 15. June 9th, General R., No. 10, doing well last August. June 11th, Mr. R. H., No. 10. July 15th, Mr. F., No. 32. August 8th, Miss O. R., No. 9. August 11th, Mr. R. No. 34; this case has been and is yet a little sensitive, Deccember 1871. August 13th, Miss T., No. 20; doing well, child about 12. September 21st, Mr. C., No. 35. October 27th, Miss H., No. 26; this case did very well, but the patient through neglect, permitted the tooth to decay on the posterior approximal surface, the pulp to become much exposed and inflamed, and insisted on having pulp devitallized; after removing the pulps, the capping could readily be seen intact on the anterior exposure. November 15th, Mrs. Eichberg, No. 5. November 25th, Miss J., No. 36 and 37. December 12th, Miss M. C., No. 3.

1871.

January 9th, Miss F. B., No. 1 and 2; a child about 11 years. January 12th, Mrs. H., No. 3. January 23d, Mrs. H., No. 4; very much decayed. January 26th, Mr. B., No. 3. February 6th, Miss C., No. 5. February 16th, Mrs. H.,

No. 6. April 5th, Capt. P., No. 7; very large, doing well. April 21st, Mr. E., No. 8. April 25th, Mr. M., No. 4. May 1st, Capt. P., No. 9. May 10th, Mrs. M., No. 9. May 13th, Mr. M., Nos. 7 and 8; cupped on both approximal surfaces of the bicuspid, doing excellently. May 16th, Mrs. C., No. 10; not seen seen since, but would have heard if there had been any trouble. May 16th, Mr. W., No. 4; this was the case of a young man about 18; the pulp was severely wounded, and I considered it a doubtful case to save the vitality, but there has been no trouble, and I can inspect it at any time, as the patient is within a few doors of me and he would assuredly come to me if there had been any pain. May 22d, Mrs. M., No. 11; this was a failure, as the pulp was not only exposed, but had been for some time, and had been in a very inflamed condition. May 24th, Mrs. L., No. 12. May 25 Miss C. M., No. 13. June 14th, Mr. S., No. 9. June 28th, Mr. C., No. 14. July 5th, Mrs. A., No. 15. July 5th, Mr. V. G., No. 15; the tooth had ached considerably, but has proved a perfect success; I see the gentleman almost daily, December 1871. July 8th, Miss L., Nos. 5 and 16. Miss S. C., No. 15. July 18th, Miss J. No. 17. July 27th, Mr. C. Nos. 16 and 18. July 28th and 31st, Miss L., Nos. 17, 21, 16, 18, 19, and 20; these teeth were badly decayed, the pulps of the central incisors exposed on both approximal surfaces, and the lateral on the anterior. The patient was a young lady of 16. I saw the teeth quite lately and find them doing well. August 8th, Mr. H. No. 9; doing well lately; patient consumptive, December 1871. August 21st Miss J., No. 22. August 23d Mr. R., No 24; doing extremely well, pulp was very much exposed. August 23d Miss B., No. 23; this case was that of a child about 11 years; nearly the whole of the dentine was gone and the pulp nearly wholly exposed on its upper sfurace, yet up to date (Dec. 15) has never given a moment's uneasiness, not even at the time of the introduction of the oxychloride. August 28th, Mrs. D., No. 9; doing very well; patient extremely delicate and nervous. September

2d, Mrs. N., No. 25; doing very well; being a rather remarkable case, having decayed below the margin of the gum; buccal surface and pulp extensively exposed. September 5th, Mrs. C., No. 4. September 9th, my own little girl, No. 23. A very large cavity, pulp almost exposed, would have been if *all* the decomposed dentine had been removed; it never has given the least trouble. September 12th, Mrs. J., No. 13. September, 21st, Mrs. T., No. 23. September 26th, Mr. G. C., No. 26. October 10th, Mr. J., No. 27. October 10th, Mrs. G. H., No. 4. October 16th, and 20th, Miss G., Nos. 23 and 28; child about 12; doing well. October 25th, Miss S. J., No. 28; temporary tooth; pulp badly exposed and aching for some time; child about 8; never troubled since filled. October 28th, Miss C. B., No. 4; temporary tooth, very analogous to the above. November 1st, Mr. D., G., No. 10. November 2d, Miss M., No. 29. November 8th, Mrs. L., No. 6; *enciente.* November 10th, Mrs. M. D., No. 30. November 11th, Miss S. J., No. 23; temporary tooth, same patient and conditions as on October 25th. November 15th, Miss L., No. 31. November 18th, Mrs. R., No. 32. November 27th, Mr. H. C., No. 8. November 30th, Mr. C., No. 33; a very difficult and doubful case on the buccal surface, cavity as large as a good-sized buck-shot, and pulp very badly exposed. Filled it without giving any pain (*during filling*); ached some little on the evening of Dec. 1st, but has not troubled since. December 13th, Mr. C., No. 20.

## Figures and Tables

**Figure f1:**